# Downregulation of FPN1 acts as a prognostic biomarker associated with immune infiltration in lung cancer

**DOI:** 10.18632/aging.202685

**Published:** 2021-03-10

**Authors:** Bing Liu, Zhiyuan Song, Yumei Fan, Guangyu Zhang, Pengxiu Cao, Danyu Li, Xiaopeng Liu, Yanzhong Chang, Ke Tan

**Affiliations:** 1 Key Laboratory of Animal Physiology, Biochemistry and Molecular Biology of Hebei Province, College of Life Sciences, Hebei Normal University, Shijiazhuang 050024, Hebei, China; 2 Department of Neurosurgery, HanDan Central Hospital, Handan 056001, Hebei, China; 3 Department of Neurosurgery, The Second Hospital of Hebei Medical University, Shijiazhuang 050000, Hebei, China

**Keywords:** FPN1, lung cancer, prognostic biomarker, immune infiltration, iron

## Abstract

Lung cancer morbidity and mortality remain the leading causes of tumor-associated death worldwide. The discovery of early diagnostic and prognostic markers of lung cancer could significantly improve the survival rate and decrease the mortality rate. FPN1 is the only known mammalian iron exporter. However, the molecular and biological functions of FPN1 in lung cancer remain unclear. Here, FPN1 mRNA expression in lung cancer was estimated using the TCGA, Oncomine, TIMER, and UALCAN databases. The prognostic role of FPN1 was evaluated using Kaplan-Meier plotter and PrognoScan. Associations between FPN1 and immune infiltration in lung cancer were evaluated by the TIMER and CIBERSORT algorithms. FPN1 mRNA and protein expressions were significantly downregulated in lung cancer. Low FPN1 expression was strongly related to worse prognosis in patients with lung cancer. GO and KEGG analyses and GSEA suggested that FPN1 was remarkably related to iron homeostasis and immunity. Importantly, FPN1 was remarkably associated with the infiltrating abundance of multiple immune cells. Moreover, FPN1 displayed a strong correlation with various immune marker sets. We investigated the clinical application value of FPN1 and provided a basis for the sensitive diagnosis, prognostication and targeted therapy of lung cancer.

## INTRODUCTION

Lung cancer is one of the leading causes of cancer-related morbidity and mortality worldwide [1]. Among various pathological types of lung cancer, NSCLC (non-small-cell lung cancer) accounts for approximately 80-85% of all lung cancer cases and is histologically divided further into three major subtypes: LCLC (large cell lung carcinoma), LUSC (lung squamous cell carcinoma) and LUAD (lung adenocarcinoma) [2]. Although the diagnosis and treatment of lung cancer have become more advanced with new targeted chemotherapy and accurate radiotherapy, improving the survival of lung cancer patients remains a challenge [2, 3]. The predictive and prognostic potential of mRNA expression has become increasingly obvious, as mRNAs have been identified as the meaningful for predicting intrinsic subtype, tumor grade and the risk of cancer recurrence [4–7].

Iron is an important trace element for multiple physiological processes, including heme synthesis, cell cycle regulation, DNA synthesis and repair and mitochondrial respiration [8–10]. Abnormal iron metabolism is frequently linked to cancer development and a poor prognosis [11, 12]. Excess iron facilitates cancer initiation, progression and metastasis, as it works as an important element for facilitating cancer cell growth and proliferation [12, 13]. It has been shown that the expression profiles of iron metabolism-related genes are altered in various cancers [14, 15].

Ferroportin 1 (FPN1), encoded by the SLC40A1 gene, is a putative multiple membrane-spanning transporter that functions as an iron exporter for nonheme iron [16–18]. FPN1 is a cell membrane protein that is ubiquitously expressed, but its expression is high in hepatocytes, duodenal enterocytes, placental syncytiotrophoblasts, and reticuloendothelial macrophages [19]. Previous studies have suggested that iron metabolism dysfunction caused by FPN1 mutations or polymorphisms is involved in hemochromatosis, inflammation, and cancer [20, 21]. Moreover, FPN1 expression is decreased in multiple cancers, including prostate cancer, ovarian cancer, breast cancer, multiple myeloma (MM) and adrenocortical carcinoma [22–28]. Reduced FPN1 mRNA expression could be utilized as a predictor of worse clinical prognosis in these types of cancer [22–29]. Downregulated FPN1 might facilitate cancer cell proliferation by reducing iron efflux. Regrettably, the expression profiles and prognostic potential of FPN1 in lung cancer are still unknown. The connection between FPN1 and immune infiltration in lung cancer remains largely unexplored.

Here, we estimated the mRNA and protein expression of FPN1 in lung cancer, examined the prognostic value of FPN1 and generated FPN1-interactive networks to investigate the mechanisms and function of FPN1. In addition, the relationship between FPN1 and the infiltrating abundance of tumor immune cells was analyzed. Our results uncovered the significant function of FPN1 in lung cancer and provide a potential connection between FPN1 and lung cancer immune infiltration and the underlying mechanism.

## RESULTS

### mRNA and protein expression of FPN1 in pancancer

**Figure 1:**
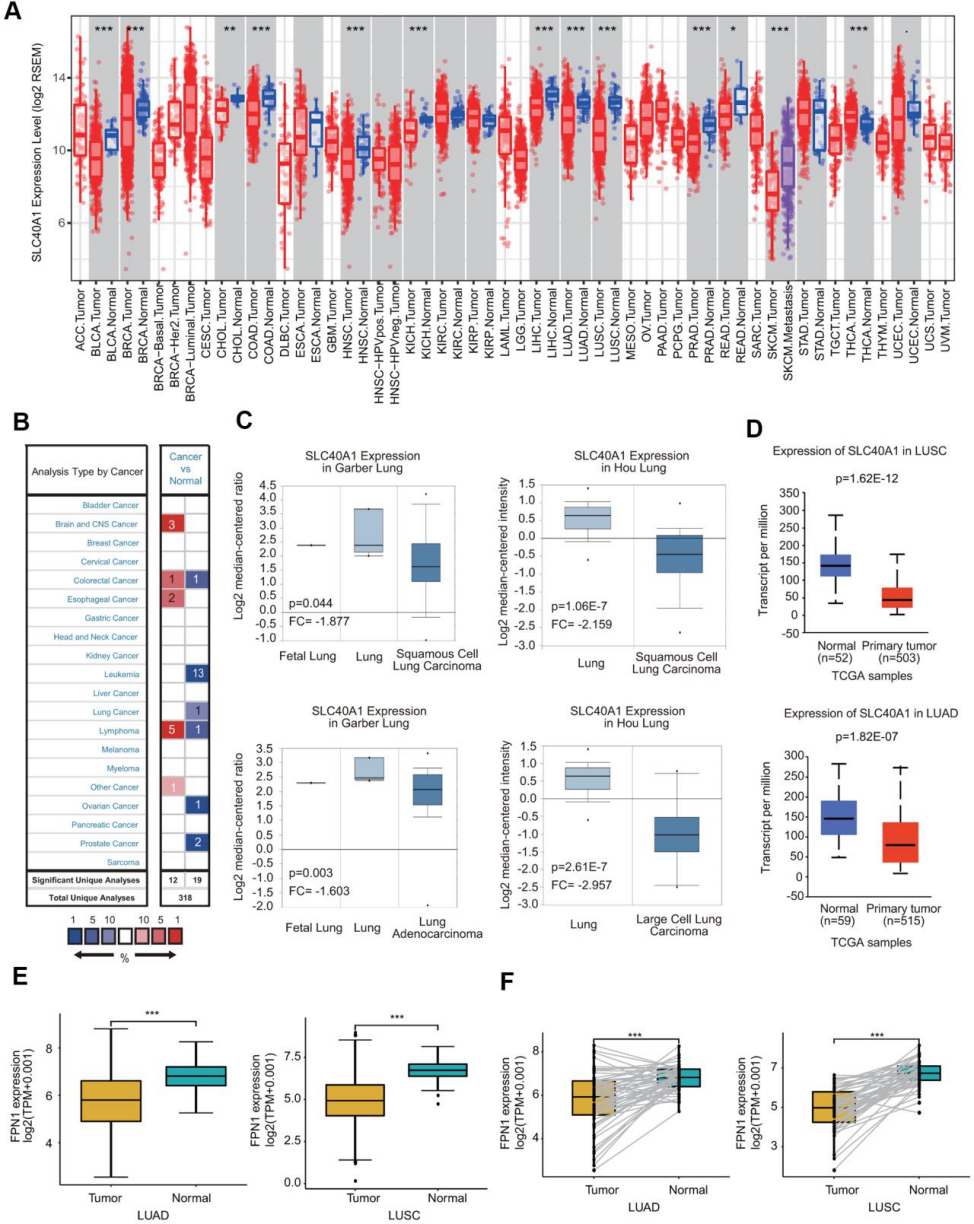
**FPN1 mRNA expression in a variety of human cancers.** (**A**) Human FPN1 expression in multiple types of cancer was determined using the TIMER database. (**B**) FPN1 expression was downregulated or upregulated in diverse cancers using the Oncomine database. (**C**) In the Garber Lung and Hou Lung datasets, the expression of FPN1 was decreased in LCLC, LUAD and LUSC tissues compared with normal tissues. (**D**) FPN1 was significantly downregulated in LUAD and LUSC in the UALCAN database. (**E**) FPN1 expression in lung cancer and adjacent normal tissues was statistically analyzed in the TCGA database. (**F**) TCGA database analysis and statistical analyses of the expression level of FPN1 were performed for 50 pairs of human lung cancer and adjacent paracancerous lung tissues. *p<0.05, **p<0.01, ***p<0.01.

We assessed FPN1 expression levels in malignant and matched paracancerous tissues using the TIMER database. The FPN1 mRNA levels in BRCA, BLCA, COAD, CHOL, HNSC, KICH, LUSC, LUAD, LIHC, PRAD, READ and SKCM were obviously decreased compared with those in their corresponding paracancerous tissues (Figure 1A). FPN1 transcriptional levels in multiple human cancers were also examined through the Oncomine online database. The database contains 31 significant, unique analyses. In 19 of the 31 unique analyses, FPN1 expression was downregulated, whereas in 12 unique analyses, FPN1 expression was upregulated compared to that in normal lung tissues. In a dataset from Garber et al., FPN1 expression levels in NSCLC, including LUSC and LUAD, were remarkably decreased compared with those in normal tissues (Figure 1C). In a dataset from Hou et al., FPN1 expression in LUSC and LCLC tissues was decreased (Figure 1C). Additionally, in a dataset by Selamat et al., FPN1 mRNA expression in LUAD was significantly reduced (Supplementary Figure 1A). The UALCAN and GEPIA databases were used to further confirm FPN1 expression in lung cancer (Figure 1D and Supplementary Figure 1B). Moreover, FPN1 expression in lung cancer and normal or paracancerous lung tissues was also confirmed through The Cancer Genome Atlas (TCGA), and the results demonstrated that FPN1 mRNA levels were greatly reduced in lung cancer tissues (Figure 1E). In addition, FPN1 expression in 50 paired lung cancer patients and normal individuals was analyzed. The mRNA level of FPN1 was consistently downregulated in both LUSC and LUAD samples (Figure 1F).

**Figure 2:**
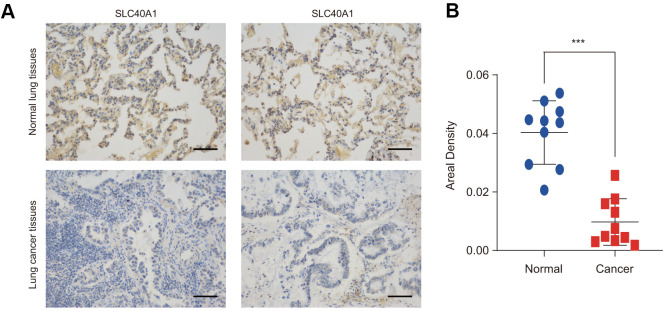
**FPN1 expression at the protein level in lung cancer patients.** (**A**) Immunohistochemical staining of FPN1 was performed in lung cancer and normal lung tissues. Representative images are shown. Scare bars, 50 μM. (**B**) The staining was quantified, as shown. The dot plot depicts the means and standard deviation of 10 images of normal lung tissues and 10 images of lung cancer patient tissues. ***p<0.001.

The protein expression level of FPN1 was further examined in lung cancer by IHC staining. As shown in Figure 2A, 2B, FPN1 protein expression was distinctly decreased in lung cancer.

### Association between FPN1 expression and the clinicopathologic parameters of lung cancer

**Figure 3:**
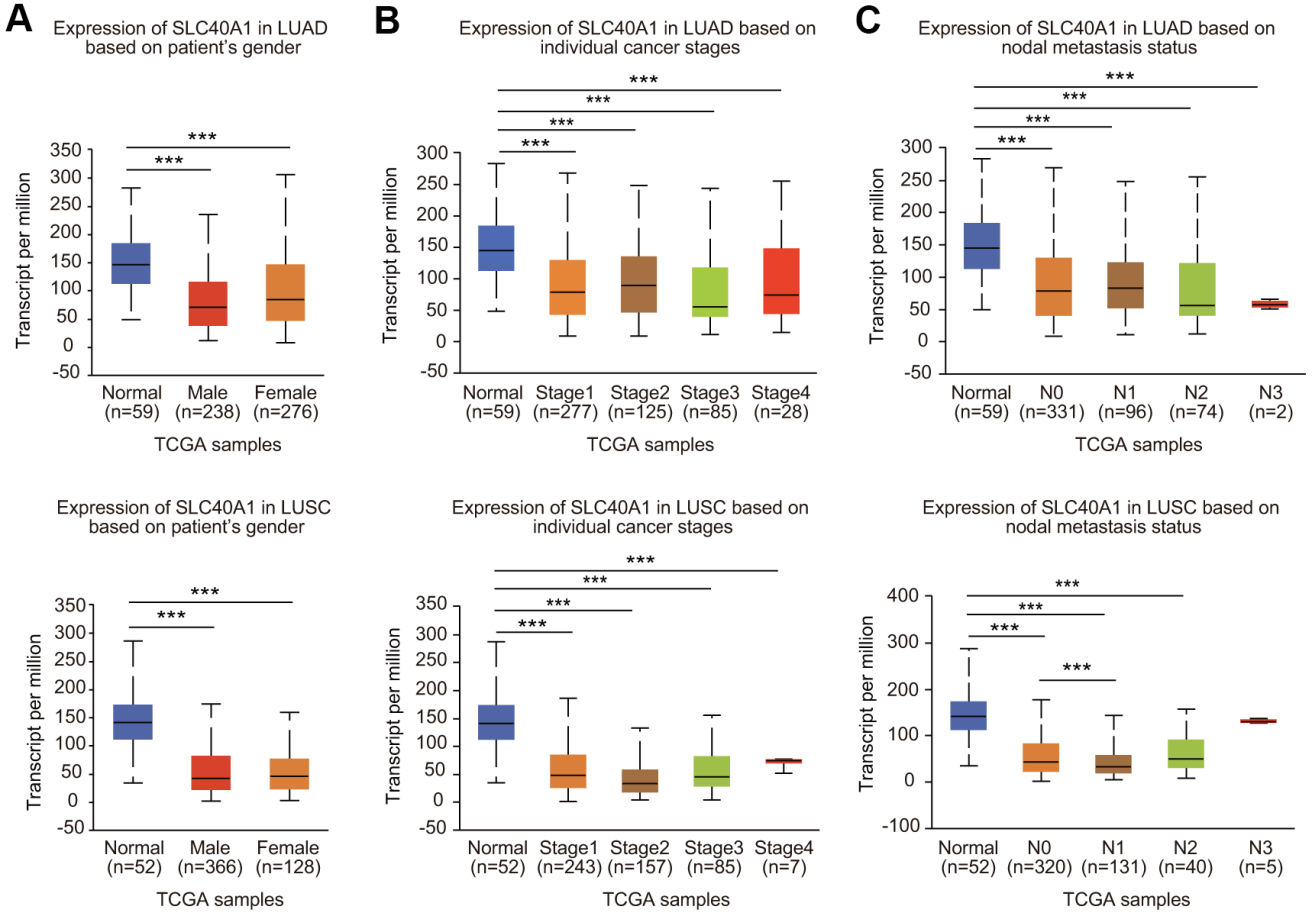
**Association between FPN1 expression and clinicopathological parameters in lung cancer patients.** The relative FPN1 expression level was determined by using the UALCAN database in (**A**) male and female lung cancer patients, (**B**) lung cancer patients with stage 1 to stage 4 diseases, and (**C**) patients with different lymph node metastatic states (from N0 to N3 based on axillary lymph node numbers).

Because FPN1 expression was greatly decreased, we next analyzed the expression profiles of FPN1 in lung cancer based on clinicopathologic parameters by using the UALCAN database [30]. As shown in Figure 3A, mining of the UALCAN database results suggested that FPN1 expression was decreased in males and females. In terms of tumor stage, significant FPN1 downregulation was observed in stages 1, 2, 3 and 4 (Figure 3B). For the nodal metastasis status, FPN1 expression levels were also apparently low in N0, N1, N2 and N3 in LUAD and LUSC (Figure 3C). FPN1 mRNA levels were decreased in lung cancer tissues from patients of different ages (Supplementary Figure 2A). FPN1 expression was significantly downregulated in LUSC patients of three different races. FPN1 expression was also dramatically decreased in Caucasian and African-American LUAD patients (Supplementary Figure 2B). Moreover, reduced FPN1 mRNA levels were shown in TP53 nonmutant and TP53-mutant lung cancer patients (Supplementary Figure 2C).

### Prognostic potential of FPN1 in lung cancer

**Figure 4:**
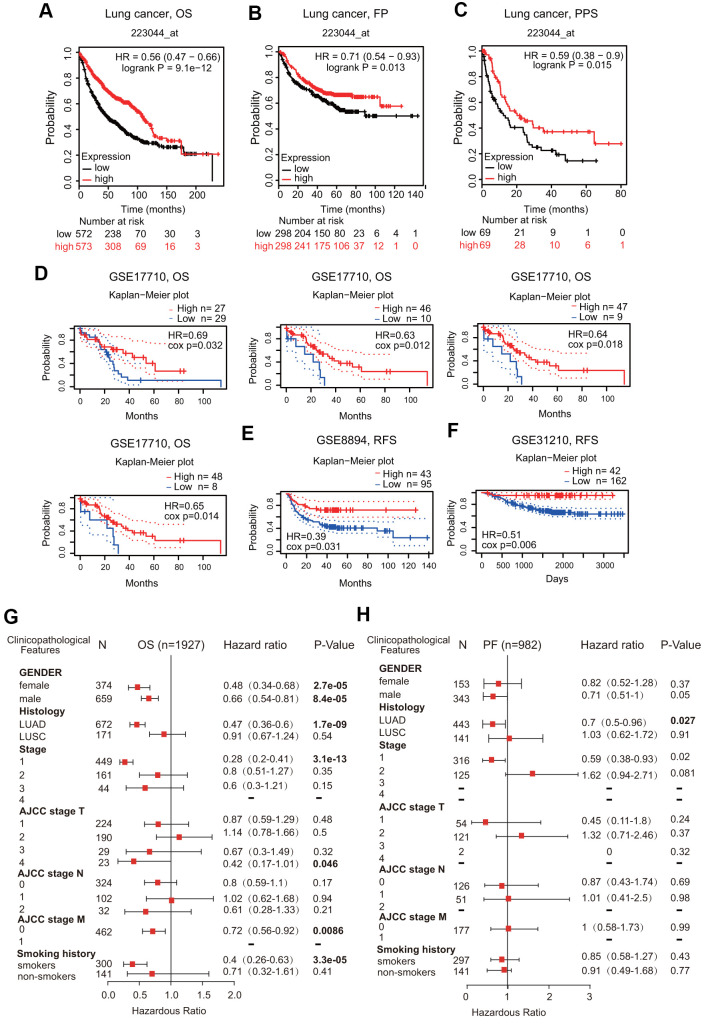
**Prognostic value of FPN1 in lung cancer.** (**A**–**C**) The correlation between FPN1 expression and OS, FP, and PPS in lung cancer patients based on Kaplan-Meier plotter. (**D**–**F**) In the PrognoScan database, the GSE17710, GSE8894 and GSE31210 cohorts were utilized to investigate the correlation between FPN1 expression and OS and RFS in lung cancer patients. (**G**, **H**) A forest plot was generated to show the connection between FPN1 expression and the clinicopathological features of LUAD and LUSC patients.

We first assessed the prognostic significance of FPN1 in various cancers. Low FPN1 expression corresponded with a poor prognosis in patients with ovarian cancer (OS, PFS and PPS) and gastric cancer (OS, FPS and PPS) (Supplementary Figure 3A, 3B). However, in breast cancer, decreased FPN1 expression corresponded with only poor OS and RFS, but it had no effect on PPS (Supplementary Figure 3C). More importantly, in lung cancer patients, decreased FPN1 expression was remarkably connected with poor OS, FPS and PPS (Figure 4A–4C).

In addition to the analysis of microarray data on FPN1 from Kaplan-Meier plotter, the prognostic potential of FPN1 was further analyzed by using the PrognoScan database. Three different cohorts (GSE17710, GSE8894 and GSE31210) including different types of lung cancer demonstrated that downregulated FPN1 mRNA expression was correlated with unfavorable OS and RFS (Figure 4D–4F).

### Prognostic potential of FPN1 according to various clinical characteristics

Low FPN1 expression was significantly linked to unfavorable OS in both female and male lung cancer patients (Figure 4G). Interestingly, FPN1 downregulation was correlated with poor OS and poor FP in LUAD patients but not in LUSC patients (Figure 4G, 4H). With respect to different tumor stages, low FPN1 expression corresponded with poor OS and poor FP only in stage 1 but not in stage 2, 3 or 4 lung cancer patients (Figure 4G, 4H). Furthermore, strong relationships between FPN1 expression and OS in AJCC stage T-4 and AJCC stage M-0 lung cancer patients were observed (Figure 4G, 4H). These findings indicate that the FPN1 mRNA expression level has prognostic value in lung cancer patients.

### Identification of key FPN1-interacting genes and proteins

**Figure 5:**
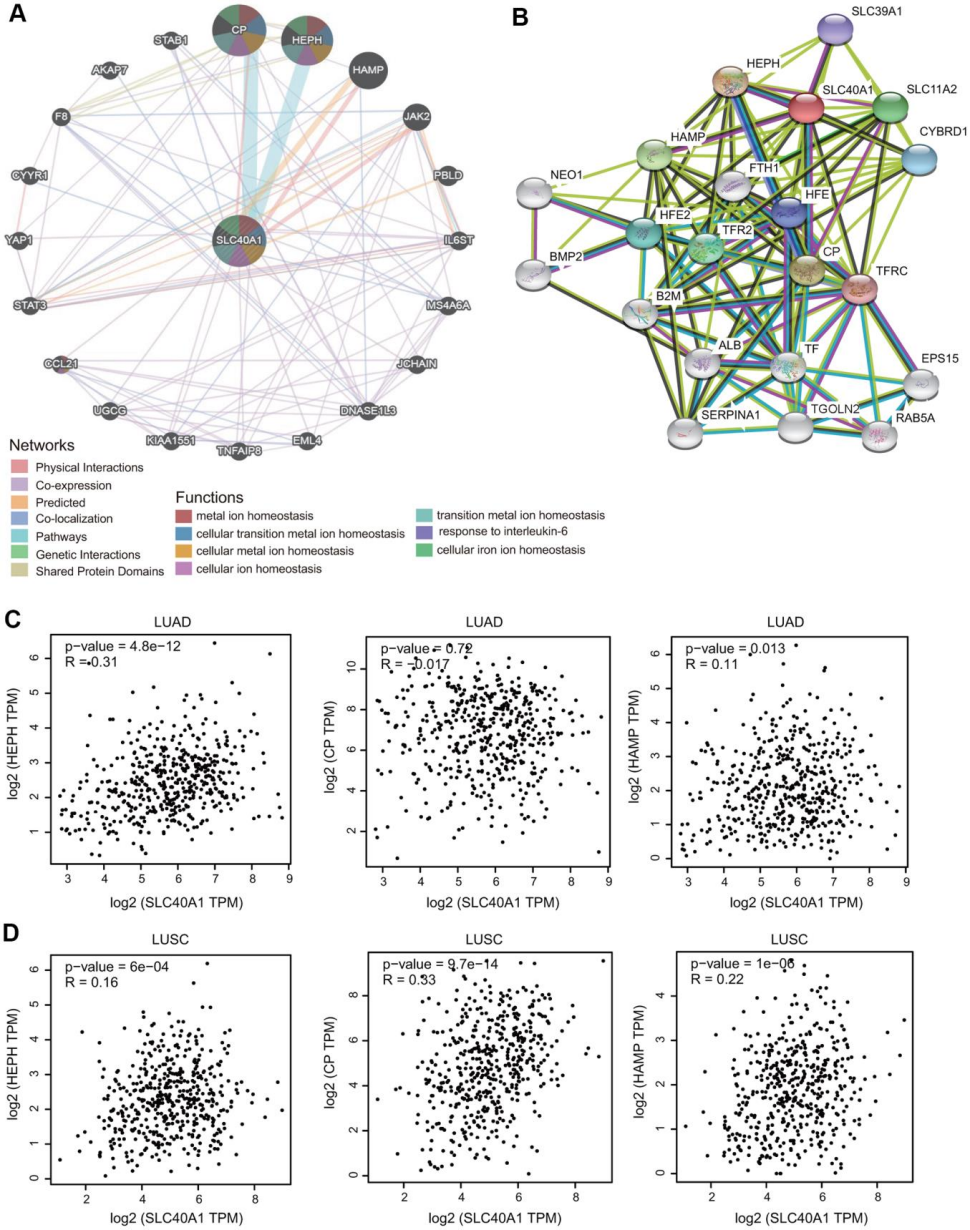
**Interaction network of FPN1.** (**A**) An interaction network for FPN1 was generated through the GeneMANIA database. (**B**) A PPI network for FPN1 was generated through the STRING database. (**C**, **D**) Scatterplots showing the correlation between FPN1 and HEPH, CP and HAMP expression in LUAD and LUSC.

First, the gene-gene interaction network for FPN1 was constructed through the GeneMANIA database [30]. The middle node represents FPN1, and the 20 surrounding nodes represent genes associated with FPN1 (Figure 5A). The five genes most significantly associated with FPN1 were ceruloplasmin (CP), hephaestin (HEPH), hepcidin (HAMP), Janus kinase 2 (JAK2) and phenazine biosynthesis-like protein domain (PBLD). Functional analysis indicated that these proteins are significantly correlated with metal ion homeostasis, cellular transition metal ion homeostasis, response to interleukin-6 and cellular iron ion homeostasis (Figure 5A). To further investigate the biological role of FPN1, a PPI network containing 21 nodes and 108 edges was generated through the STRING online database (Figure 5B). The genes of the 5 most significant nodes were HEPH, CP, HAMP, solute carrier family 11 member 2 (SLC11A2) and transferring receptor 2 (TFR2) (Figure 5B).

Additionally, we identified three genes from both the STRING and GeneMANIA databases: CP, HAMP and HEPH. The relationship between FPN1 and these hub proteins was evaluated in GEPIA, and the Pearson correlation coefficients were calculated. As shown in Figure 5C, 5D, FPN1 was positively coexpressed with HEPH and HAMP in LUAD and with CP, HEPH and HAMP in LUSC.

### Pathways regulated by FPN1 in LUAD and LUSC identified by gene ontology (GO) and kyoto encyclopedia of genes and genomes (KEGG) enrichment analyses

**Figure 6:**
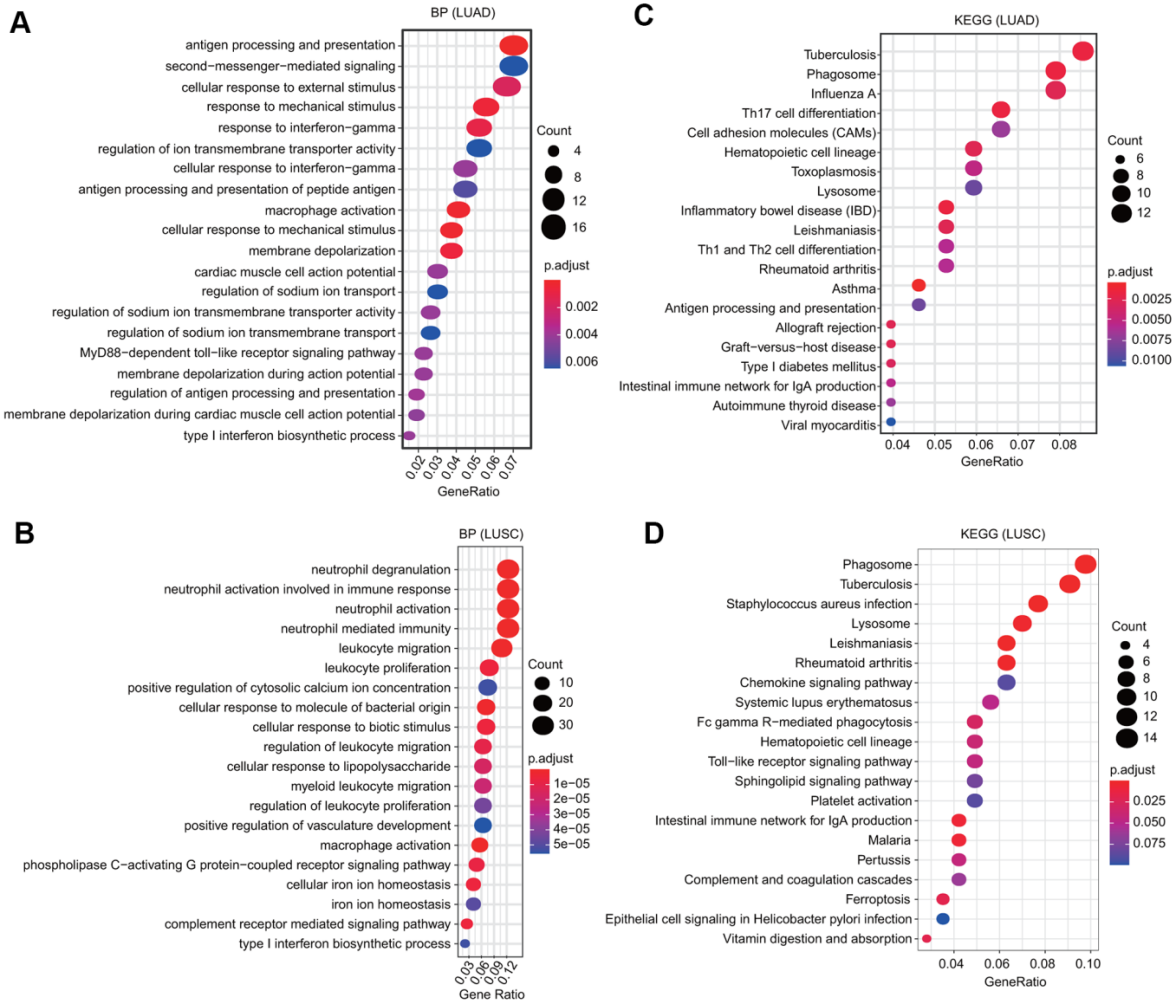
**GO and KEGG analyses of FPN1 in lung cancer.** (**A**, **B**) GO analyses of the biological function of FPN1 in LUAD and LUSC. (**C**, **D**) KEGG analyses in LUAD and LUSC. The count represents the number of genes associated with enriched GO or KEGG pathways. The color represents the -log10-transformed P-value.

We selected the first 300 genes that were positively and significantly associated with the FPN1 gene and performed GO and KEGG analyses through the clusterProfiler package [30]. GO terms can be classified into three categories: molecular function (MF), biological process (BP) and cellular component (CC) [30]. Bubble plots representing the top 20 enriched BF, MF and CC terms of GO analysis were constructed (Figure 6A and Supplementary Figure 4). Regarding the BP terms, the results showed that in LUAD, the terms regulation of ion transmembrane transporter activity and membrane depolarization were associated with FPN1; in LUSC, the terms cellular iron ion homeostasis and complement receptor mediated signaling pathway were associated with FPN1 (Figure 6A, 6B). In addition, we found that several immune pathways were highly correlated with FPN1, including antigen processing and presentation, the cellular response to interferon-gamma, the response to interferon-gamma, antigen processing and presentation of peptide antigen, macrophage activation, and type I interferon biosynthetic process in LUAD; and neutrophil degranulation, neutrophil activation, neutrophil activation involved in the immune response, macrophage activation, neutrophil-mediated immunity, leukocyte migration, leukocyte proliferation, and type I interferon biosynthetic process in LUSC (Figure 6A, 6B). KEGG pathway analysis also revealed that FPN1 was associated with immune response-related terms, such as Th17 cell differentiation, inflammatory bowel disease, Th1 and Th2 cell differentiation, the intestinal immune network for IgA production in LUAD and Staphylococcus aureus infection, the intestinal immune network for IgA production, the chemokine signaling pathway, Fc gamma R-mediated phagocytosis, and epithelial cell signaling in Helicobacter pylori infection in LUSC (Figure 6C, 6D).

### Pathways regulated by FPN1 identified by gene set enrichment analysis (GSEA)

**Figure 7:**
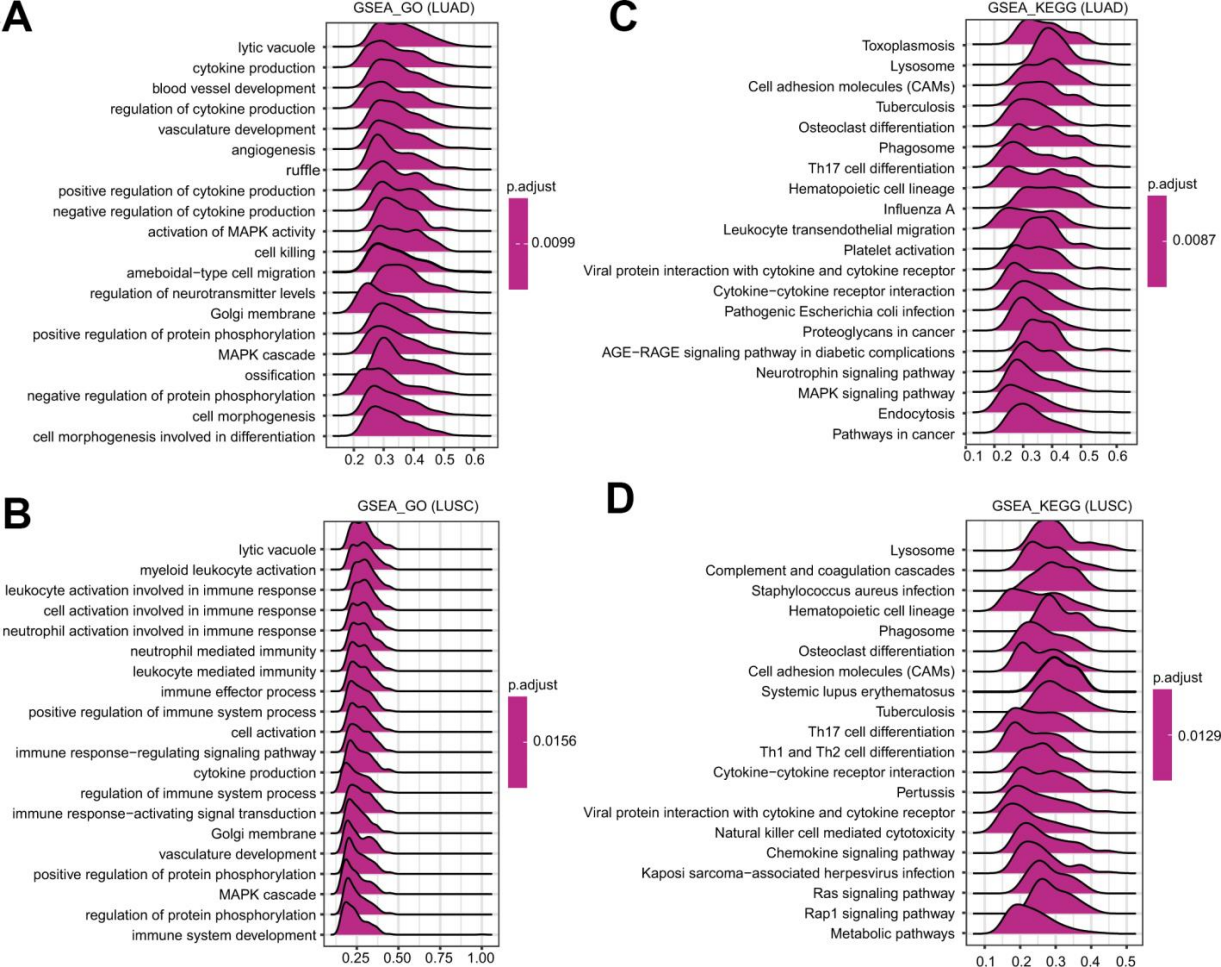
**GSEA of FPN1 in lung cancer.** (**A**, **B**) Merged plots were constructed to exhibit the enriched pathways correlated with FPN1 in LUAD according to GO and KEGG analyses. (**C**, **D**) Merged plots were constructed to exhibit the enriched pathways correlated with FPN1 in LUSC according to GO and KEGG analyses.

To further identify the potential mechanisms affected by FPN1 in LUAD and LUSC, GSEA was performed to estimate signaling pathways affected by FPN1 in lung cancer. As shown in Figure 7A, 7B, among the GO terms, the top 20 signaling pathways influenced by FPN1 were mainly enriched in immune-related activities, such as cytokine production, regulation of cytokine production and positive/negative regulation of cytokine production in LUAD and in leukocyte activation involved in immune response, myeloid leukocyte activation, cell activation involved in immune response, neutrophil activation involved in immune response, neutrophil mediated immunity, immune effector process, leukocyte mediated immunity, immune response-regulating signaling pathway, positive regulation of immune system process, cytokine production, regulation of immune system process, immune response-activating signal transduction, and immune system development in LUSC. Similarly, among the KEGG terms, GSEA revealed multiple immune functional gene sets that were enriched in both LUAD and LUSC, including gene sets related to cytokine-cytokine receptor interactions, Th17 cell differentiation and viral protein interactions with cytokines and cytokine receptors (Figure 7C, 7D). These results revealed that FPN1 might be a potential indicator of the status of the tumor microenvironment.

### Correlation analysis between FPN1 expression and major types of infiltrating immune cells

**Figure 8:**
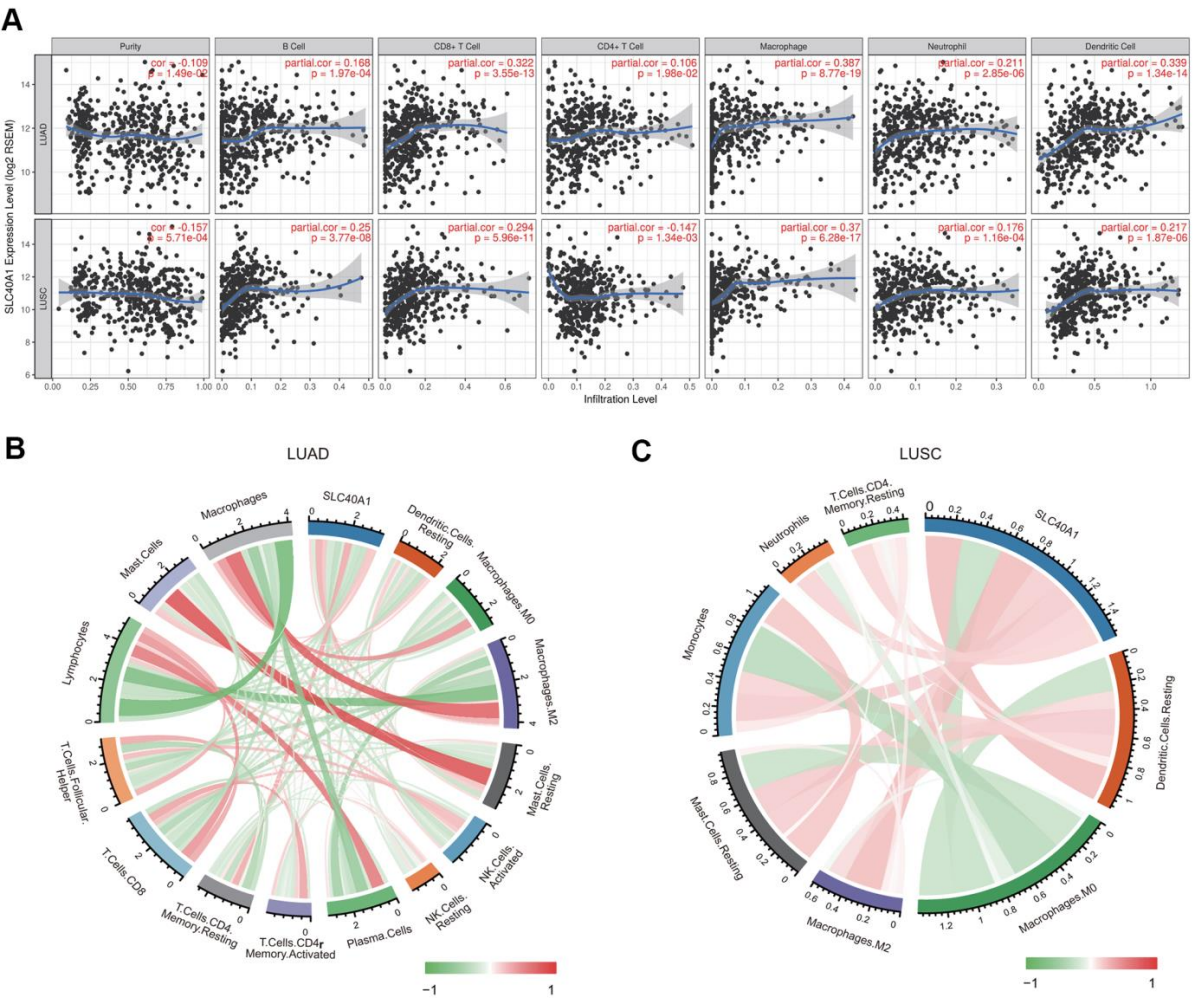
**Association between FPN1 and immune cell infiltration in lung cancer.** (**A**) FPN1 showed a significant correlation with the infiltrating abundance of CD4+ T cells, CD8+ T cells, B cells, neutrophils, macrophages and dendritic cells using the TIMER database. (**B, C**) The relationship between FPN1 and the infiltrating abundance of different immune cells was investigated using CIBERSORT. All results are shown in a circos plot.

We then evaluated the relationship between FPN1 and diverse tumor-infiltrating immune cells. The results from the TIMER database suggested that FPN1 was remarkably correlated not only with tumor purity but also with the infiltrating levels of different immune cells, including CD4+ T cells, CD8+ T cells, B cells, neutrophils, macrophages and dendritic cells, in LUAD and LUSC (Figure 8A). These results suggest that FPN1 is tightly connected with the infiltration of immune cells in lung cancer, especially the infiltration of macrophages and CD8+ T cells.

We also assessed the relationship between FPN1 and immune cell infiltration through CIBERSORT, which is an established computational resource. Notably, FPN1 expression was positively and remarkably linked with the infiltrating levels of memory CD4 resting T cells, M2 macrophages, mast cells, macrophages, resting mast cells, monocytes, and dendritic cell but negatively linked with the infiltrating levels of plasma cells, lymphocytes, follicular helper T cells, CD8 T cells, activated NK cells, resting NK cells, M0 macrophages, and activated memory CD4 T cells in LUAD (Figure 8B and Supplementary Table 1). Moreover, FPN1 was positively and significantly linked with the infiltrating levels of M2 macrophages, resting dendritic cells, monocytes, resting mast cells, neutrophils, resting CD4 memory T cells, and dendritic cells but linked with the infiltrating levels of M0 macrophages, regulatory T (Treg) cells, plasma cells, activated mast cells, activated NK cells, and naïve CD4 memory T memory cells in LUSC (Figure 8C and Supplementary Table 2).

### Relationship between FPN1 and distinct immune marker sets

As previous studies reported [4, 5], the genes listed in Table 1 were used to characterize different immune cells. As shown in Table 1 and Figure 8, FPN1 expression was remarkably correlated with the levels of most markers in different types of immune cells in LUSC and LUAD.

**Table 1:** Relationship between FPN1 and gene marker sets of different immune cells using the TIMER database.

**Description**	**Gene markers**	**LUAD**				**LUSC**			
		**None**		**Purity**		**None**		**Purity**	
		**Cor**	**p**	**Cor**	**p**	**Cor**	**p**	**Cor**	**p**
**B cell**	CD19	0.045	0.305	0.001	0.987	0.136	**	0.066	0.152
	CD79A	-0.01	0.816	-0.055	0.223	0.176	***	0.111	*
**T cell (general)**	CD3D	0.154	***	0.122	**	0.223	***	0.163	***
	CD3E	0.192	***	0.169	***	0.209	***	0.149	**
	CD2	00227	***	0.206	***	0.238	***	0.185	***
**CD8+ T cell**	CD8A	0.128	**	0.108	*	0.242	***	0.196	***
	CD8B	0.096	*	0.075	0.0974	0.215	***	0.183	***
**Monocyte**	CD86	0.329	***	0.321	***	0.367	***	0.344	***
	CSF1R	0.361	***	0.352	***	0.337	***	0.306	***
**TAM**	CCL2	0.183	***	0.152	***	0.211	***	0.176	***
	CD68	0.335	***	0.327	***	0.419	***	0.407	***
	IL10	0.345	***	0.336	***	0.393	***	0.367	***
**M1**	IRF5	0.215	***	0.192	***	0.081	0.0692	0.066	0.151
	PTGS2	0.017	0.692	0.017	0.0707	0.106	*	0.078	0.0898
**M2**	CD163	0.263	***	0.245	***	0.374	***	0.338	***
	VSIG4	0.33	***	0.319	***	0.451	***	0.426	***
	MS4A4A	0.397	***	0.392	***	0.444	***	0.419	***
**Neutrophils**	CEACAM8	0.373	***	0.368	***	0.099	*	0.092	*
	ITGAM	0.332	***	0.313	***	0.159	***	0.098	*
	CCR7	0.262	***	0.237	***	0.148	***	0.085	0.0643
**Natural killer cell**	KIR2DL1	-0.056	0.208	-0.064	0.158	0.111	*	0.092	*
	KIR2DL3	-0.009	0.833	-0.036	0.425	0.168	***	0.142	**
	KIR2DL4	-0.165	***	-0.183	***	0.192	***	0.164	***
	KIR3DL1	-0.014	0.76	-0.04	0.376	0.185	***	0.147	**
	KIR3DL2	-0.031	0.485	-0.055	0.221	0.119	**	0.093	*
	KIR3DL3	-0.118	**	-0.129	**	0.08	0.0743	0.068	0.139
**Dendritic cell**	HLA-DPB1	0.451	***	0.459	***	0.314	***	0.272	***
	HLA-DQB1	0.317	***	0.305	***	0.262	***	0.213	***
	HLA-DRA	0.472	***	0.475	***	0.354	***	0.319	***
	HLA-DPA1	0.482	***	0.49	***	0.348	***	0.315	***
	CD1C	0.467	***	0.448	***	0.352	***	0.32	***
	NRP1	0.174	***	0.164	***	0.328	***	0.296	***
	ITGAX	0.155	***	0.127	**	0.159	***	0.099	*

**Figure 9:**
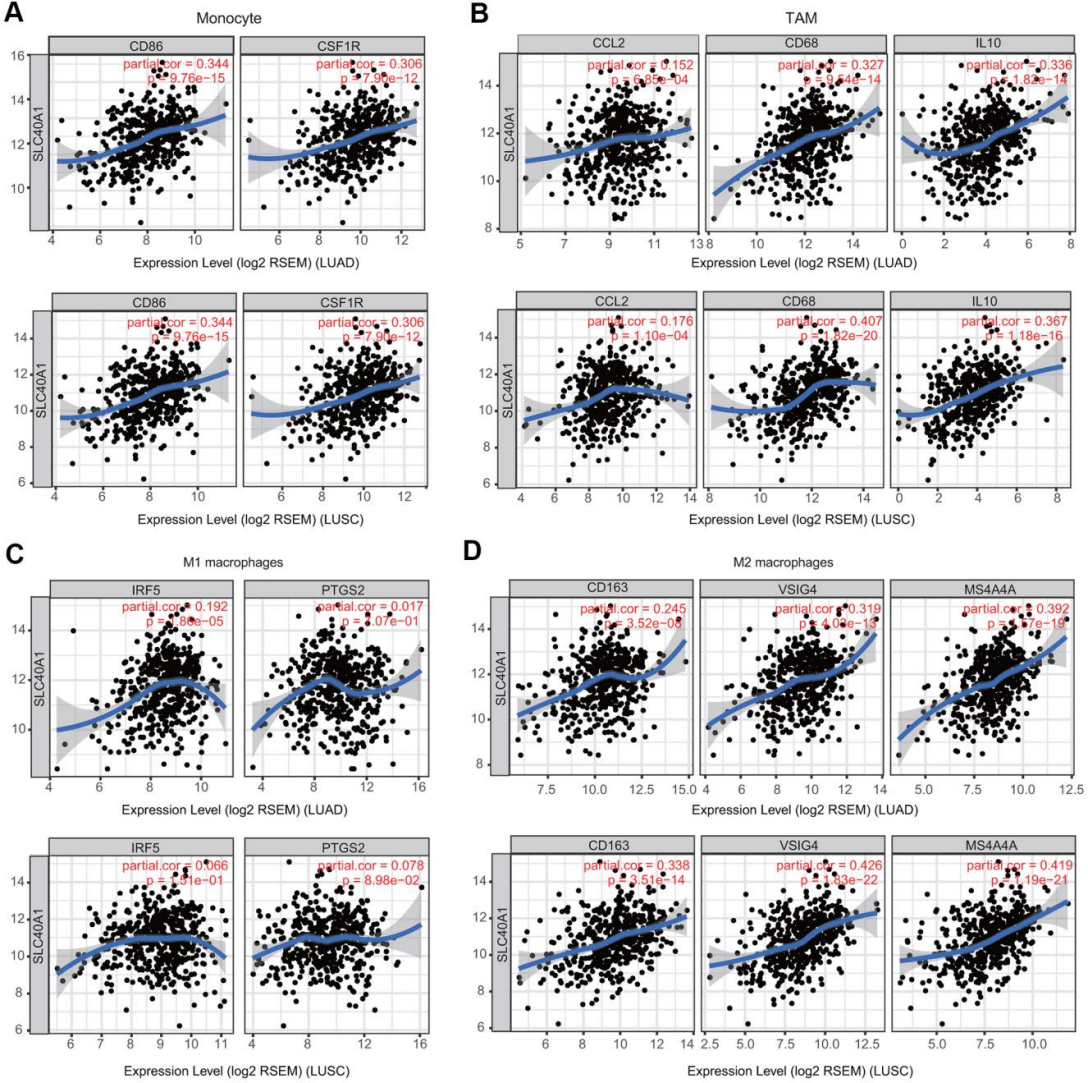
**Association of FPN1 with macrophage polarization in LUAD and LUSC.** Relationship between FPN1 and various gene markers of (**A**) monocytes, (**B**) TAMs, (**C**) M1 macrophages and (**D**) M2 macrophages in LUAD and LUSC.

Because macrophages are the immune cell type that is most strongly correlated with FPN1 expression (Figure 9), we further investigated the connections between FPN1 and immune marker sets of monocytes, tumor-associated macrophages (TAMs), M1 macrophages and M2 macrophages through GEPIA [30]. FPN1 exhibited a positive significant correlation with TAM infiltration in LUAD and LUSC tissues but not in corresponding normal lung tissues (Table 2).

**Table 2:** Relationship between FPN1 and gene marker sets of monocytes and macrophages using the GEPIA database.

**Description**	**Gene markers**	**LUAD**				**LUSC**			
		**Tumor**		**Normal**		**Tumor**		**Normal**	
		**R**	**P**	**R**	**P**	**R**	**P**	**R**	**P**
**Monocyte**	CD86	0.4	***	-0.12	0.36	0.37	***	0.22	0.12
	CSF1R	0.42	***	-0.0014	0.99	0.33	***	0.17	0.25
**TAM**	CCL2	0.24	***	-0.15	0.24	0.2	***	-0.35	*
	CD68	0.44	***	-0.27	*	0.42	***	0.018	0.9
	IL10	0.43	***	-0.067	0.61	0.38	***	0.0068	0.96
**M1 macrophage**	NOS2	0.1	*	0.31	*	0.05	0.27	0.075	0.6
	IRF5	0.28	***	-0.24	0.064	0.088	0.051	-0.011	0.9
	PTGS2	0.09	*	-0.22	0.092	0.12	**	-0.25	0.085
**M2 macrophage**	CD163	0.26	***	-0.32	*	0.32	***	-0.14	0.34
	VSIG4	0.38	***	-0.25	0.057	0.43	***	-0.023	0.87
	MS4A4A	0.46	***	-0.19	0.14	0.43	***	0.14	0.32

We also estimated the connection between FPN1 and various T cells (Table 3) [30]. By using the TIMER database, we found that FPN1 was associated with 34 of 36 T cell markers in LUAD and with 31 of 36 T cell markers in LUSC (Table 3). Moreover, after adjusting for tumor purity, FPN1 was remarkably associated with 33 of 36 markers of T cells in LUAD and with 23 of 36 markers of T cells in LUSC (Table 3).

**Table 3:** Relationship between FPN1 and gene marker sets of diverse T cells using the TIMER database.

**Description**	**Gene markers**	**LUAD**				**LUSC**			
		**None**		**Purity**		**None**		**Purity**	
		**Cor**	**p**	**Cor**	**p**	**Cor**	**p**	**Cor**	**p**
**Th1 cell**	TBX21	0.129	**	0.095	*	0.129	**	0.068	0.136
	STAT4	0.331	***	0.322	***	0.273	***	0.237	***
	STAT1	0.103	*	0.081	0.0713	0.191	***	0.158	***
	TNF	0.173	***	0.124	**	0.006	0.9	-0.07	0.128
**Th1-like cell**	HAVCR2	0.341	***	0.333	***	0.375	***	0.348	***
	CXCR3	0.152	***	0.121	**	0.195	***	0.138	**
	BHLHE40	0.125	**	0.099	*	-0.084	0.0604	-0.126	**
	CD4	0.405	***	0.41	***	0.341	***	0.308	***
**Th2 cell**	STAT6	0.209	***	0.209	***	0.007	0.879	-0.009	0.854
	STAT5A	0.293	***	0.273	***	0.144	**	0.084	0.0684
**Treg cell**	FOXP3	0.129	**	0.093	*	0.092	*	0.027	0.563
	CCR8	0.258	***	0.238	***	0.178	***	0.128	**
	TGFB1	0.31	***	0.302	***	0.033	0.461	-0.016	0.722
**Resting Treg cell**	FOXP3	0.129	**	0.093	*	0.092	*	0.027	0.563
	IL2RA	0.165	***	0.137	**	0.286	***	0.239	***
**Effector Treg cell**	FOXP3	0.129	**	0.093	*	0.092	*	0.027	0.563
	CCR8	0.258	***	0.238	***	0.178	***	0.128	**
	TNFRSF9	0.154	***	0.117	**	0.208	***	0.16	***
**Effector T cell**	CX3CR1	0.538	***	0.533	***	0.431	***	0.405	***
	FGFBP2	0.313	***	0.288	***	0.077	0.0835	0.109	*
	FCGR3A	0.275	***	0.268	***	0.394	***	0.367	***
**Naïve T cell**	CCR7	0.262	***	0.237	***	0.148	***	0.085	0.0643
	SELL	0.33	***	0.314	***	0.314	***	0.282	***
**Effector memory T cell**	DUSP4	-0.297	***	-0.291	***	0.116	**	0.074	0.108
	GZMK	0.266	***	0.248	***	0.221	***	0.162	***
	GZMA	0.099	*	0.072	0.112	0.285	***	0.239	***
**Resident memory T cell**	CD69	0.358	***	0.355	***	0.292	***	0.244	***
	CXCR6	0.227	***	0.213	***	0.3	***	0.25	***
	MYADM	0.138	**	0.137	**	0.113	*	0.062	0.177
**General memory T-cell**	CCR7	0.262	***	0.237	***	0.148	***	0.085	0.0643
	SELL	0.33	***	0.314	***	0.314	***	0.282	***
	IL7R	0.359	***	0.396	***	0.232	***	0.19	***
**Exhausted T cell**	HAVCR2	0.341	***	0.333	***	0.375	***	0.348	***
	LAG3	-0.074	0.0917	-0.112	*	0.095	*	0.047	0.302
	CXCL13	0.053	0.231	0.01	0.832	0.15	***	0.09	*
	LAYN	0.182	***	0.161	***	0.113	*	0.113	*

### Prognostic value of FPN1 according to immune cells in LUAD

**Figure 10:**
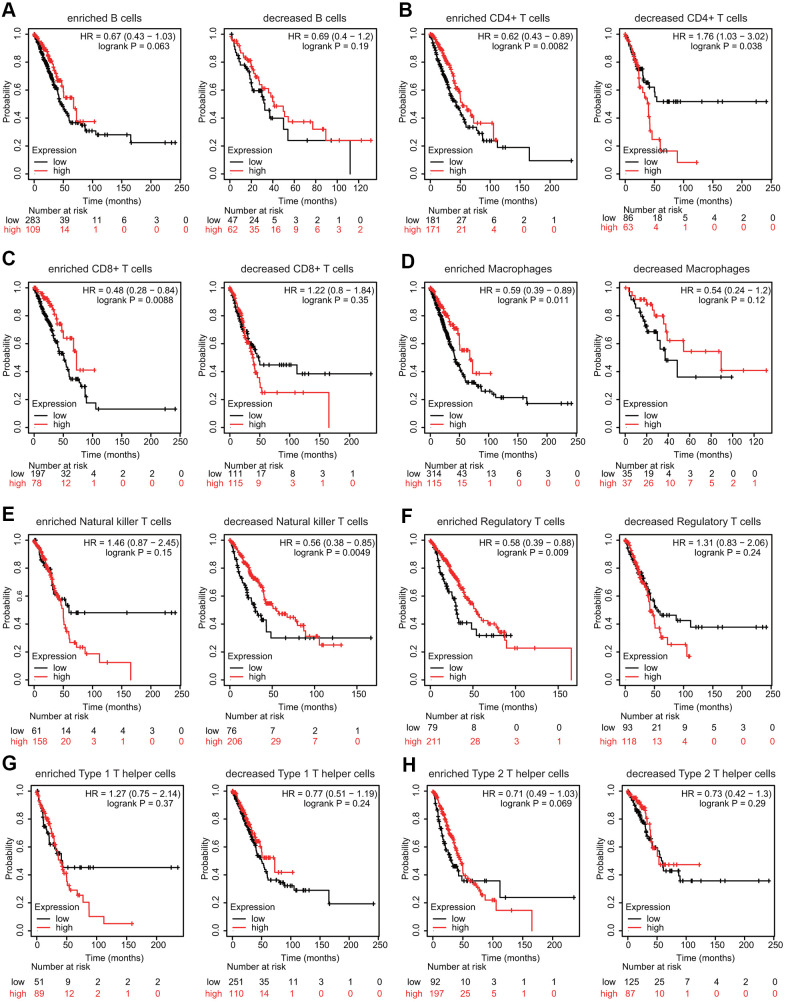
**Effects of FPN1 on survival based on multiple immune cell subgroups in LUAD.** (**A**–**H**) Associations of FPN1 and OS in diverse immune cell subgroups in LUAD patients.

We then explored whether FPN1 expression influenced the prognosis of LUAD patients by directly affecting immune cell infiltration. Prognostic analyses based on FPN1 expression in LUAD in different immune cell subgroups were performed. Low expression of FPN1 in the enriched memory CD4+ T cell, enriched CD8+ T cell, enriched macrophage, decreased natural killer (NK) T cell, and enriched regulatory T cell (Treg) cohorts in LUAD was associated with poor prognosis (Figure 10B–10F). However, there was no significant association between low/high FPN1 expression and LUAD patient prognosis in the B cell, type 1 T helper cell, or type 2 T helper cell cohorts (Figure 10A, 10G, 10H).

## DISCUSSION

Tumor cells need increased amounts of iron for their rapid growth and proliferation [10, 11]. Iron plays different roles in cancer cells under different conditions. On the one hand, iron can alter the cellular redox status as an electron donor for free radicals. Excess free radicals will promote gene mutations that may accelerate tumor initiation. On the other hand, iron is essential for cancer cell growth and proliferation as a kind of nutrient element [11, 12]. Cancer cells show an iron-addicted phenotype caused by abnormal expression of iron metabolism-related genes [31]. Many kinds of iron chelators, including deferasirox (DFX), deferoxamine (DFO) and Dp44mT, have been developed as anticancer drugs that target iron metabolism [32]. Iron-chelating agents usually trigger apoptotic cell death in various cancer cells. Several iron chelators also induce other types of cell death, such as ferroptosis. For example, sulfasalazine can cause ferroptosis in cancer cells by inhibiting xCT [33]. In clinical studies, the potential benefit of iron-chelating agents in tumor inhibition was obtained in patients with leukemia and neuroblastoma [34]. In addition, Dp44mT could significantly reverse drug resistance to etoposide in breast cancer cells and vinblastine in epidermal carcinoma cells [35]. In a clinical study, DFX was used to treat a patient with high serum ferritin and negiltuzumab ozoomidine-resistant leukemia [36]. DFX lowers serum ferritin, eliminating the requirement for continuous blood transfusions and helping patients achieve complete remission [36]. DFO has also been demonstrated to effectively overcome multidrug resistance in leukemia cells by triggering apoptosis, reducing intracellular iron concentrations and downregulating the expression of MDR1 [37]. Moreover, recent advances in molecular biology indicate that iron chelators can be used in combination with molecular targeted drugs to treat intractable and drug resistant cancers.

To satisfy the demand for high iron, cancer cells change their iron metabolism not only by enhancing the uptake of iron and adjusting iron storage, but also by decreasing the export of iron. The iron efflux system controlled by FPN1 is one of the important molecular mechanisms used to adjust the iron contents in cells and tissues [17–20]. FPN1 was identified simultaneously as an iron export protein from three different groups [16–18]. The human SLC40A1 gene is located on chromosome 2-q and contains 8 exons spanning over 20 kb [16–18]. FPN1 is distributed throughout a wide range of cells, including duodenal enterocytes, placental trophoblasts, macrophages, hepatocytes and central nervous system cells. The expression of FPN1 is normally regulated by HAMP, which binds to FPN1 and then induces its degradation [38, 39]. HAMP is a 25-amino acid iron regulatory hormone that is mainly generated by hepatocytes [40]. Binding of HAMP to FPN1 results in internalization and proteolysis of FPN1, followed by a reduction in iron export from target cells and consequently an increase in cellular iron levels [38, 39]. Although the iron export ability of FPN1 has been well established, recent reports have indicated that FPN1 also regulates the cellular levels of manganese (Mn). FPN1-overexpressing Xenopus oocytes export more Mn than normal oocytes [41]. In HEK293T cells, inducible expression of FPN1 decreases Mn accumulation and cytotoxicity [42]. Interestingly, disease mutations affect the function of FPN1 in regulating Mn concentration and the stability of FPN1 [21]. Ferroportin disease, also known as type IV hereditary hemochromatosis (HH), is primarily caused by missense mutations in FPN1 [43–45]. HH comprises heterogeneous iron homeostasis disorders caused by genetic factors. Currently, numerous FPN1 heterozygous mutations have been identified to be associated with type IV HH [20]. In addition, due to the increased need for phenotypic and genetic testing, rare SLC40A1 variants have been found by chance in patients with secondary causes of hyperferritinemia. More importantly, FPN1 is closely involved in oncogenesis. Several literatures have demonstrated that FPN1 expression is reduced in prostate cancer, breast cancer, ovarian cancer, MM and adrenocortical carcinoma [23–28]. In addition, FPN1 overexpression reduces the growth of xenografted breast cancer cells *in vivo* [22, 25]. Moreover, a reduction in FPN1 expression level is strongly linked with unfavorable prognosis in breast cancer and adrenocortical carcinoma [25, 28]. A decrease in FPN1 expression on the cell surface triggers increased cellular iron levels and is associated with the emergence of aggressive phenotypes. Therefore, FPN1 may also have many unknown functions and influence the pathological processes of different diseases. An in-depth study of the molecular function of FPN1 will provide a new direction for understanding the pathogenesis and treatment of diseases.

With in-depth research, there is a growing realization that FPN1 expression is mediated by posttranslational, posttranscriptional, and transcriptional regulatory mechanisms. FPN1 expression is mediated at the posttranscriptional level by the famous iron-regulatory protein/iron-responsive element (IRP/IRE) pathway [8, 9]. There is an IRE sequence in the 5’-UTR of FPN1 transcripts. IRP proteins bind to IRE and reduce translational efficiency in response to iron deprivation [8–12, 38, 46]. At the transcriptional level, nuclear factor erythroid 2-like 2 (NRF2) can directly modulate FPN1 expression to affect cancer cell proliferation by regulating intracellular iron and ROS accumulation [47, 48]. In addition, Sirtuin 2 (Sirt2) maintains cellular iron concentrations through the deacetylation of NRF2, which results in decreased FPN1 expression in cancer cells and in a mouse model [49]. Moreover, myeloid zinc-finger-1 (MZF-1) inhibits prostate cancer growth by upregulating FPN1 expression [50]. Under iron deficiency, hypoxia-inducible factor 2α (HIF-2α) directly binds to the FPN1 promoter and induces its expression [51]. Although FPN1 profoundly influences cellular iron levels and is essential for systemic iron trafficking, the biological effects of abnormal FPN1 expression on lung cancer development and the connection between FPN1 and immune infiltration remain largely unexplored.

In this study, we extracted data from lung cancer patients from different clinical databases, including TIMER, Oncomine, UALCAN and TCGA. The transcriptional level of FPN1 was downregulated in lung cancer tissues (Figure 1). We also analyzed the expression of FPN1 according to diverse clinical characteristics, such as sex, age, tumor stage, histological grade and distant metastasis, via the UALCAN database (Figure 2 and Supplementary Figure 2). FPN1 expression was remarkably reduced in all tumor stages and correlated with axillary lymph node metastasis. Moreover, the effect of FPN1 expression on the survival of cancer patients was analyzed. Low FPN1 expression levels were remarkably connected with poor prognosis in LUAD and LUSC patients (Figure 3). The relationships between FPN1 and OS and PFS based on different clinical parameters of lung cancer patients were also evaluated. Our findings emphasize an important role of FPN1 in tumorigenesis and cancer progression in lung cancer.

Recent studies have demonstrated that cancer progression and recurrence are promoted not only by genetic alterations but also by the TME [52]. Immune cells in the TME are associated with tumor progression and recurrence. Moreover, the effects of infiltrating immune cells on clinical outcomes have been widely recognized. T cell checkpoint inhibitors have shown significant improvement in the treatment of multiple types of cancer. PD-1 and CTLA-4 inhibitors exhibit promising anticancer effects in multiple cancers, including NSCLC [53]. Recently, a study found that a significant alteration in T cells and NK cells was induced in stage I LUAD lesions [54]. Moreover, FPN1 is primarily expressed in iron-recycling macrophages. TAMs have been indicated to inhibit T cells from recognizing and killing tumor cells. An analysis of the immune microenvironment of hepatocellular carcinoma by single-cell RNA sequencing analyses indicated that TAMs correlated with prognosis [55]. An enrichment of TAM gene signatures was obviously associated with worse survival in lung cancer patients, suggesting tumor-infiltrating TAMs as potential therapeutic targets. Interestingly, TAM-like macrophages in hepatocellular carcinoma highly express two genes: FPN1 and GPNMB [55]. In addition, the loss of FPN1 obviously increases the secretion of well-known cytokines, including IL-6 and TNF-α, in mouse macrophages [56]. However, reduced FPN1 mitigated inflammatory responses in macrophages in response to Salmonella infection [57]. These findings imply that FPN1 is also involved in immune regulation.

Another nonnegligible finding from our study is that FPN1 is remarkably correlated with the immune response and immune infiltration levels in lung cancer. We performed GO analysis, KEGG analysis and GSEA to explore the mechanism of FPN1 in lung cancer (Figures 5, 6). The results indicate that FPN1 is important for iron ion homeostasis and significantly associated with the immune response. Furthermore, we used TIMER, GEPIA and CIBERSORT to uncover the connection between FPN1 and immune cell infiltration in lung cancer for the first time. Our findings suggest a significant relationship between FPN1 expression and immune status in the TME of LUAD and LUSC (Figures 7, 8 and Tables 1–3). Among the six types of immune cells, FPN1 strongly affected the infiltrating levels of CD8+ T cells and macrophages. In addition, FPN1 was positively correlated with multiple subtypes of T cells, including memory T, effector T, Th1-like, effector Treg and exhausted T cells. FPN1 may modulate tumor immune cell infiltration by regulating intracellular iron levels in the tumor microenvironment. Moreover, we found that FPN1 may affect the prognosis of patients with LUAD through effects on immune infiltration (Figure 9). Our results also imply that FPN1 is able to recruit immune cells or mediate immune cell infiltration in NSCLC. Nevertheless, the precise functions of FPN1 in the tumor immune microenvironment still need further in-depth exploration.

In summary, FPN1 expression is significantly decreased in lung cancer, and FPN1 may act as an early-stage diagnostic biomarker. Decreased FPN1 expression levels are correlated with unfavorable prognosis in lung cancer. Furthermore, FPN1 may affect the cancer-associated immune response and immune cell infiltration in both LUAD and LUSC. Therefore, FPN1 may act as a meaningful diagnostic and sensitive prognostic marker and immunity-associated therapeutic target for lung cancer. Further studies are required to confirm these results and to explore the mechanisms and immunoregulatory functions of FPN1 in lung cancer.

## MATERIALS AND METHODS

### Oncomine database

The Oncomine database (http://www.oncomine.org), which includes 715 datasets and 86,733 samples, was utilized to analyze the mRNA levels of FPN1 in lung cancer and normal or paracancerous tissues. Our search was performed based on the following criteria: P-value < 0.05, fold change < -1.5, and gene ranking all.

### GEPIA database

GEPIA (http://gepia.cancer-pku.cn), a mining online database, pulls data from the UCSC Xena server. We utilized the GEPIA database to investigate FPN1 mRNA expression in lung cancer and the association between FPN1 and the expression levels of candidate genes.

### UALCAN database

The UALCAN database (http://ualcan.path.uab.edu/) provides comprehensive cancer transcriptome and clinical patient data (pulled from TCGA). We evaluated the expression level of FPN1 to compare it not only between lung cancer and corresponding paracancerous tissues but also across various subgroups stratified by sex, pathological stage, tumor grade and other clinicopathological parameters.

### TIMER database

TIMER is an interactive and user-friendly online tool that can be used to systematically evaluate the expression of gene sets related to infiltrating immune cells in data from TCGA. In the present study, the connection between FPN1 expression and immune cell infiltration in LUSC and LUAD was analyzed. Moreover, associations between FPN1 and gene markers of diverse tumor-infiltrating immune cells were investigated through TIMER.

### Kaplan-Meier plotter analysis

The relationships between the expression level of FPN1 and prognosis (i.e., overall survival [OS], median time to first progression survival [FPS], relapse-free survival [RFS] and postprogression survival [PPS]) of cancer patients were examined with Kaplan-Meier plotter (http://kmplot.com). In addition, the prognostic potential of FPN1 based on multiple clinicopathological features was also analyzed with Kaplan–Meier plotter [30].

### PrognoScan database

PrognoScan (http://www.prognoscan.org/) was employed to investigate the prognostic significance of FPN1 in lung cancer patients [30]. Red curves correspond to high FPN1 expression, and blue curves correspond to low FPN1 expression.

### Interaction network analysis

In this study, the GeneMANIA online database (http://www.genemania.org) was utilized to generate the FPN1 interaction network and to evaluate the roles of these interactions. STRING was utilized to generate a PPI network of FPN1 (https://string-db.org/) [30].

### GO terms and KEGG analyses

GO analysis was applied to verify the roles and pathways of FPN1 in lung cancer. GO analysis, producing results consisting of MF, BP and CC terms, as well as KEGG were performed with the R package clusterProfiler [30].

### Gene set enrichment analysis (GSEA)

GSEA was performed to explore the underlying mechanisms of FPN1. We employed the clusterprofiler package in R to analyze the gene sets.

### CIBERSORT estimation

The CIBERSORT algorithm (https://cibersort.stanford.edu/), an online platform, was used to identify the effect of FPN1 on fractions of immune cells based on bulk samples from the LUAD and LUSC cohorts. Spearman’s correlation test and a P-value <0.05 were conducted to explore the relationship between FPN1 and the infiltration levels of different immune cells.

### Immunohistochemistry (IHC) staining

The study was approved by the Institutional Research Ethics Committee of HanDan Central Hospital. Written informed consent was obtained from the participants. Ten paraffin-embedded lung cancer samples and normal lung samples were used for IHC staining. Briefly, 4-μm sections of tissues were slightly mounted on glass slides, deparaffinized in xylene, and then rehydrated in sequentially increasing dilutions of alcohol. Antigen retrieval was carried out using the water-bath heating method. The sections were cooled and rinsed, and endogenous peroxidase activity was quenched by incubation in 3% hydrogen peroxide. Then, the sections were washed three times with PBS, incubated with calf serum to block nonspecific antigens for 10 min, incubated with polyclonal rabbit anti-FPN1 antibody (1:100, D163909, Sangon Biotech, Shanghai, China) at room temperature (RT) for 1 h, washed with PBS three times, and then incubated with secondary antibody at RT for 40 min. Dried sections were observed with an optical microscope. A semiquantitative integration method was employed to analyze the staining intensity.

### Statistical analysis

PrognoScan and Kaplan-Meier plotter were employed to generate survival curves. Moreover, Spearman’s correlation, Pearson’s correlation and statistical significance analyses were used to assess the correlation between the expression of genes [30]. P < 0.05 was considered statistically significant.

## Supplementary Material

Supplementary Figures

Supplementary Table 1

Supplementary Table 2
