# Supplementary material for: Downregulation of FPN1 acts as a prognostic biomarker associated with immune infiltration in lung cancer
Source: Aging (Albany NY). 2021 Mar 10;13(6):8737–61. doi: 10.18632/aging.202685 (PMC8034901; doi:10.18632/aging.202685)
Supplement: Supplementary Figures [file aging-13-202685-s001.pdf]

SUPPLEMENTARY FIGURES

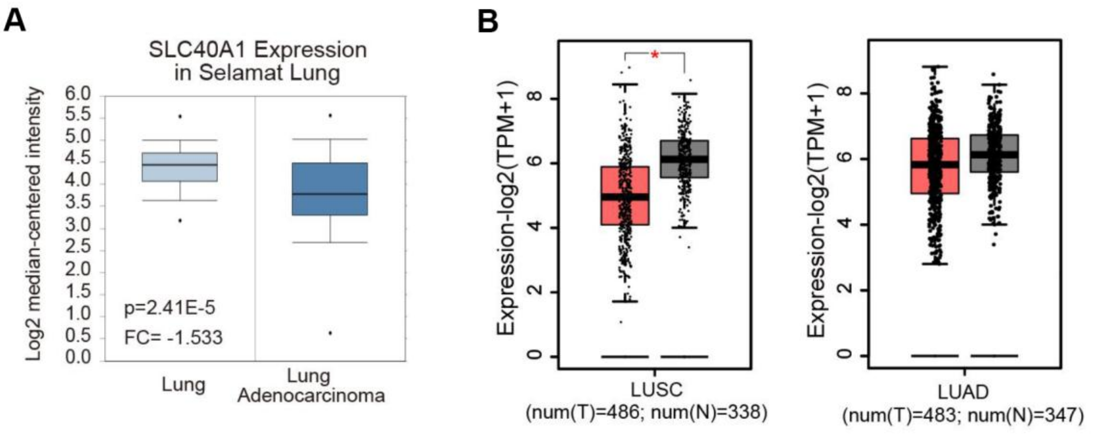

Supplementary Figure 1. FPN1 expression level in lung cancer. (**A**) In the Selamat Lung dataset, FPN1 transcriptional levels were decreased in LUAD tissues. (**B**) FPN1 was significantly downregulated in LUAD and LUSC in the GEPIA database.

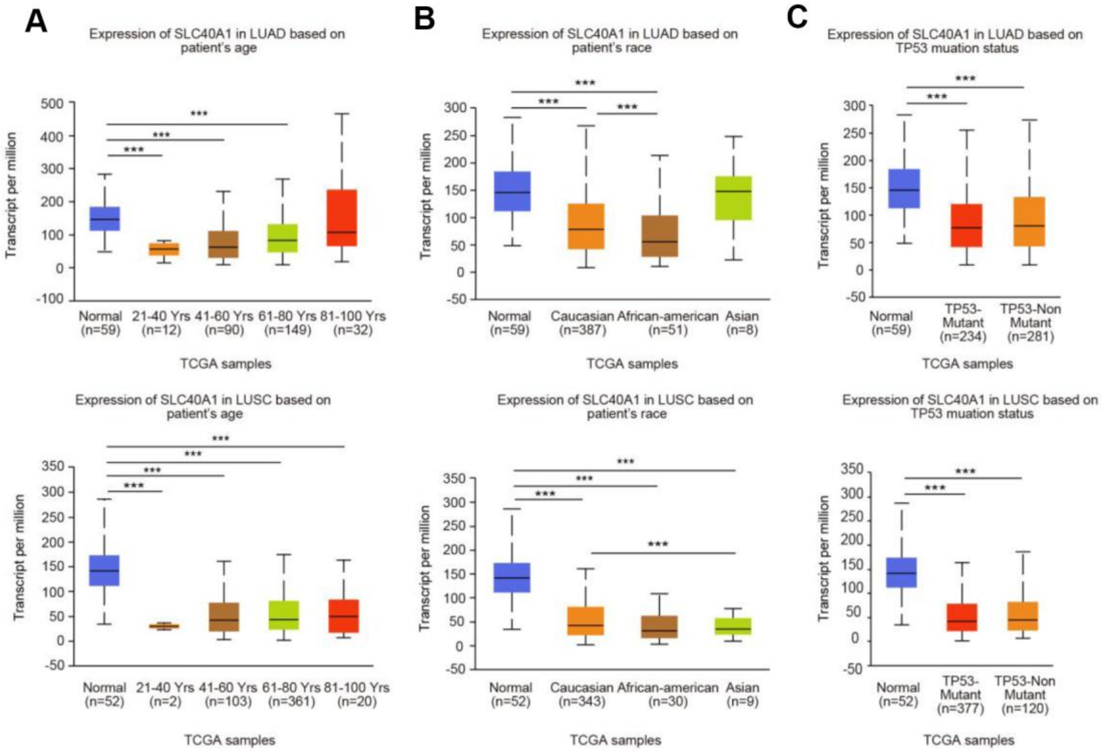

Supplementary Figure 2. Relative FPN1 expression was analyzed by using the UALCAN database in (**A**) lung cancer patients of different ages (from stage 21 to 100), (**B**) patients of different races and (**C**) patients with different TP53 mutation statuses.

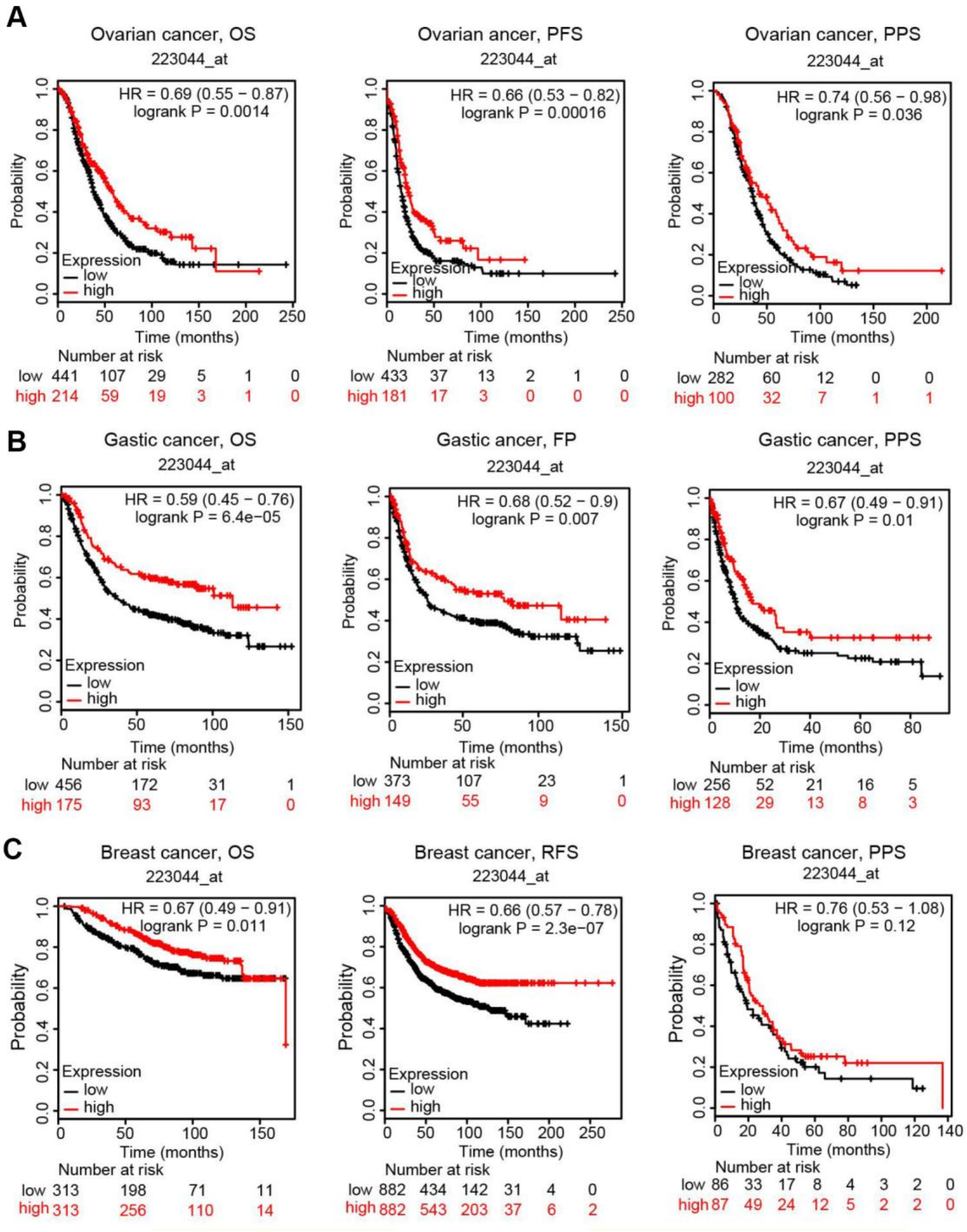

Supplementary Figure 3. Survival analyses of FPN1 in several human cancers. The correlations between FPN1 expression and OS, PFS and PPS in ovarian cancer patients (**A**); OS, FPS and PPS in gastric cancer patients (**B**); and OS, RFS and PPS in breast cancer patients (**C**) were examined through Kaplan-Meier plotter.

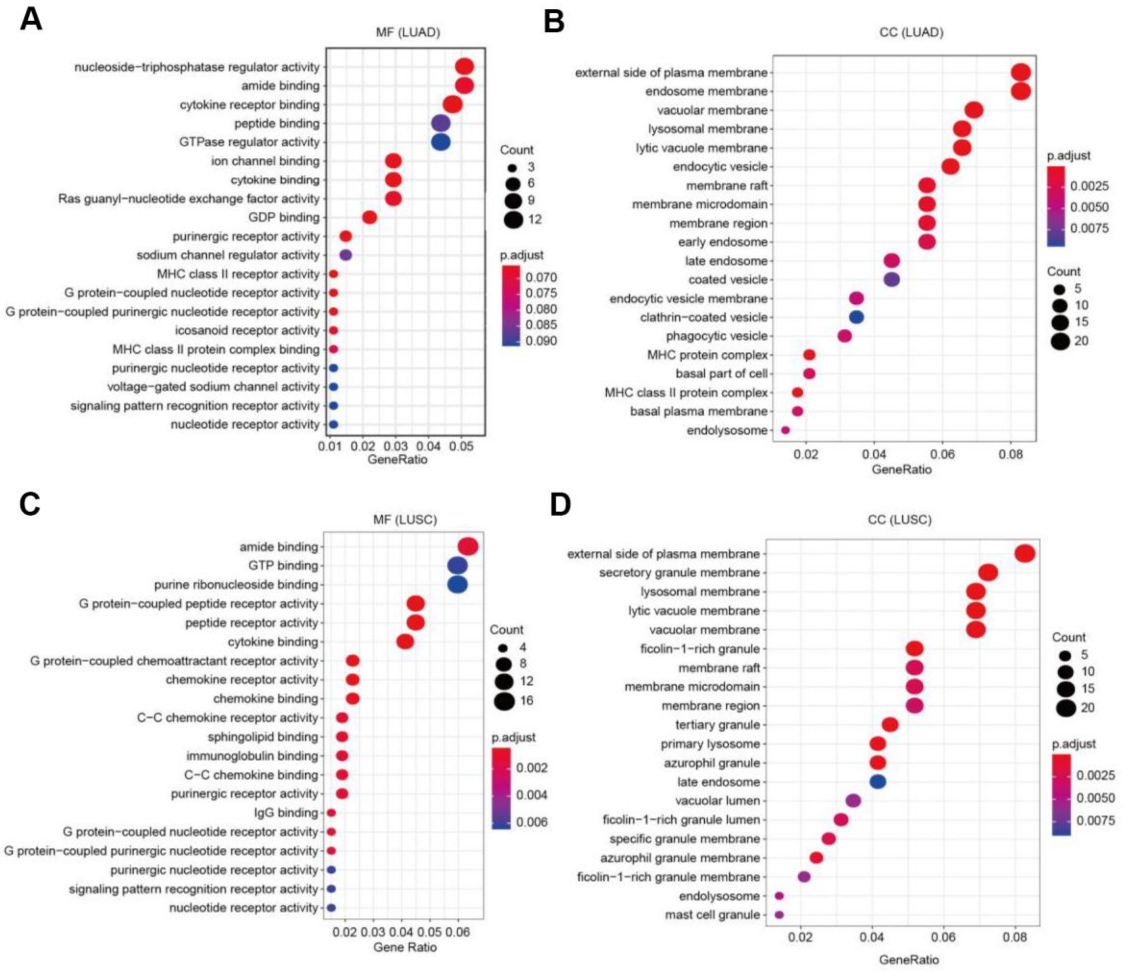

Supplementary Figure 4. GO enrichment analyses of FPN1 in lung cancer. (**A**, **B**) GO analyses of the molecular function and cellular component terms of FPN1 in LUAD. (**C**, **D**) GO analyses of the molecular function and cellular component terms of FPN1 in LUSC.
